# A240 THE PROTECTIVE EFFECT OF *HYMENOLEPIS DIMINUTA* INFECTION IN EXPERIMENTAL COLITIS CAN BE INDEPENDENT OF IL-4RΑ SIGNALING

**DOI:** 10.1093/jcag/gwad061.240

**Published:** 2024-02-14

**Authors:** L Kraemer Rocha, S Li, S Rajeev, A Wang, D McKay

**Affiliations:** University of Calgary, Calgary, AB, Canada; University of Calgary, Calgary, AB, Canada; University of Calgary, Calgary, AB, Canada; University of Calgary, Calgary, AB, Canada; University of Calgary, Calgary, AB, Canada

## Abstract

**Background:**

Helminth parasites are potent inducers of Th2 immunity, characterized by the production of IL-4, IL-5, IL-13 and IL-10, and mobilization of regulatory cells. This orchestrated immune response establishes an immunomodulatory and/or immunosuppressive environment that may be protective against colitis and other inflammatory diseases. Earlier studies reveal that infection with the tapeworm parasite *Hymenolepis diminuta* ameliorates chemical-induced colitis in immunocompetent mice, and highlighted participation of the anti-inflammatory cytokine IL-10 in the mechanisms of protection. Moreover, the IL-4ra/STAT6 signaling pathway plays a pivotal role in promoting the Th2 immune response that is crucial for parasite expulsion: IL-4ra KO mice cannot expel the worm. However, the need for activation of IL-4rasignaling in the suppression of colitis by *H. diminuta* infection is unknown.

**Aims:**

To determine if the inhibition of DNBS-induced colitis evoked by infection with *H. diminuta* requires IL-4ra signaling.

**Methods:**

IL-4ra KO mice (n=4-8/group) were orally infected with 5 viable cysticercoids of *H. diminuta* and 8 days post-infection (dpi) were treated with 2.5 mg of dinitrobenzene sulfonic acid (DNBS). Subsequently, mice were assessed 72h post-DNBS. Disease was assessed by macroscopic disease activity score, colon length, weight loss, MPO activity of the colon, and leucocyte profile by blood count. Production of IL-10 by concanavalin-A (con-A; 48h)-stimulated splenocytes was measured by ELISA.

**Results:**

An increase in blood eosinophilia in *H. diminuta* infected mice indicated a successful infection. DNBS treatment induced acute colitis in IL-4ra KO mice, and surprisingly prior infection with *H. diminuta* resulted in significantly less severe colitis, as evidenced by reduction in macroscopic disease activity score, prevention of weight loss, reduced MPO colon levels and maintenance of regular colon length. However, splenocytes from *H. diminuta*-infected IL-4ra KO mice did not show increased IL-10 production evoked by con-A compared to cells from non-infected mice.

**Conclusions:**

These results indicate that the protection against DNBS-induced colitis by prior infection with *H. diminuta* can occur in the absence of IL-4ra signalling. This suggests redundancy in the anti-colitic effect of infection with this helminth parasite, such that absence of IL4ra is compensated for via another, yet to be determined, mechanism. We speculate that this may be via participation of type 2 innate lymphoid cell activity, mobilization of regulatory T cells or innate immunity: all possibilities that need to be rigorously tested.

**Funding Agencies:**

CIHRBeverly Phillips Rising Star and Cumming School of Medicine (U. Calgary) Fellowships

